# Community-Acquired Pneumonia in Patients With Diabetes: Narrative Review

**DOI:** 10.2196/82215

**Published:** 2026-03-10

**Authors:** Yun Xie, Ao Zhang, Ying Wang, Ruilan Wang

**Affiliations:** 1 Department of Critical Care Medicine, Shanghai General Hospital, Shanghai Jiao Tong University School of Medicine, Shanghai, 201620, China, +86 13917138008; 2 Department of Critical Care Medicine, Shanghai General Hospital of Nanjing Medical University, Shanghai, China; 3 Shanghai Institute of Immunology, Department of Immunology and Microbiology, Key Laboratory of Cell Differentiation and Apoptosis of Chinese Ministry of Education, Shanghai Jiaotong University School of Medicine, Shanghai, 200025, China

**Keywords:** diabetes mellitus, community-acquired pneumonia, pathophysiology, pathogens, complications, treatment, prevention, microbiome

## Abstract

**Background:**

Patients with diabetes carry a 1.5- to 2-fold higher risk of community-acquired pneumonia (CAP) and experience more severe outcomes, yet the mechanisms that integrate metabolic dysregulation, pathogen shifts, and novel cell death pathways remain fragmented.

**Objective:**

This study aimed to synthesize current evidence on epidemiology, pathophysiology, causative pathogens, clinical outcomes, and management of CAP in adults with diabetes and to identify research gaps for future trials.

**Methods:**

A narrative review (1999 to August 2025) of PubMed, EMBASE, the Cochrane Library, and Web of Science was conducted. GRADE (Grading of Recommendations Assessment, Development, and Evaluation) was used to rate evidence from 81 selected English-language studies (randomized controlled trials, cohorts, and meta-analyses).

**Results:**

Diabetes increases CAP incidence (relative risk 1.73, 95% CI 1.46‐2.04), hospitalization (+30%‐50%), and 30-day mortality (odds ratio 1.67, 95 % CI 1.45-1.92). Key drivers include hyperglycemia-induced immune paralysis, pulmonary microangiopathy, ferroptosis, glycation and methylation changes, and gut-lung dysbiosis that collectively favor multidrug-resistant Gram-negative bacilli (*Klebsiella* and *Pseudomonas*) and severe viral and fungal coinfections. Host-targeted therapy with moderate glycemic control (5‐10 mmol/L), continued metformin, and pathogen-directed antibiotics improves survival, whereas single-dose PCV20 and annual influenza vaccination prevents approximately 45% of CAP admissions. Emerging strategies (nanozymes, ferroptosis inhibitors, probiotics, and proteolysis-targeting chimeras) are still preclinical.

**Conclusions:**

CAP in patients with diabetes is a distinct, more severe entity mediated by metabolic-immune crosstalk. Multicenter randomized controlled trials integrating tight glucose monitoring, novel host-directed agents, and microbiome modulation are warranted to translate mechanistic insights into better outcomes.

## Introduction

**Figure 1.**
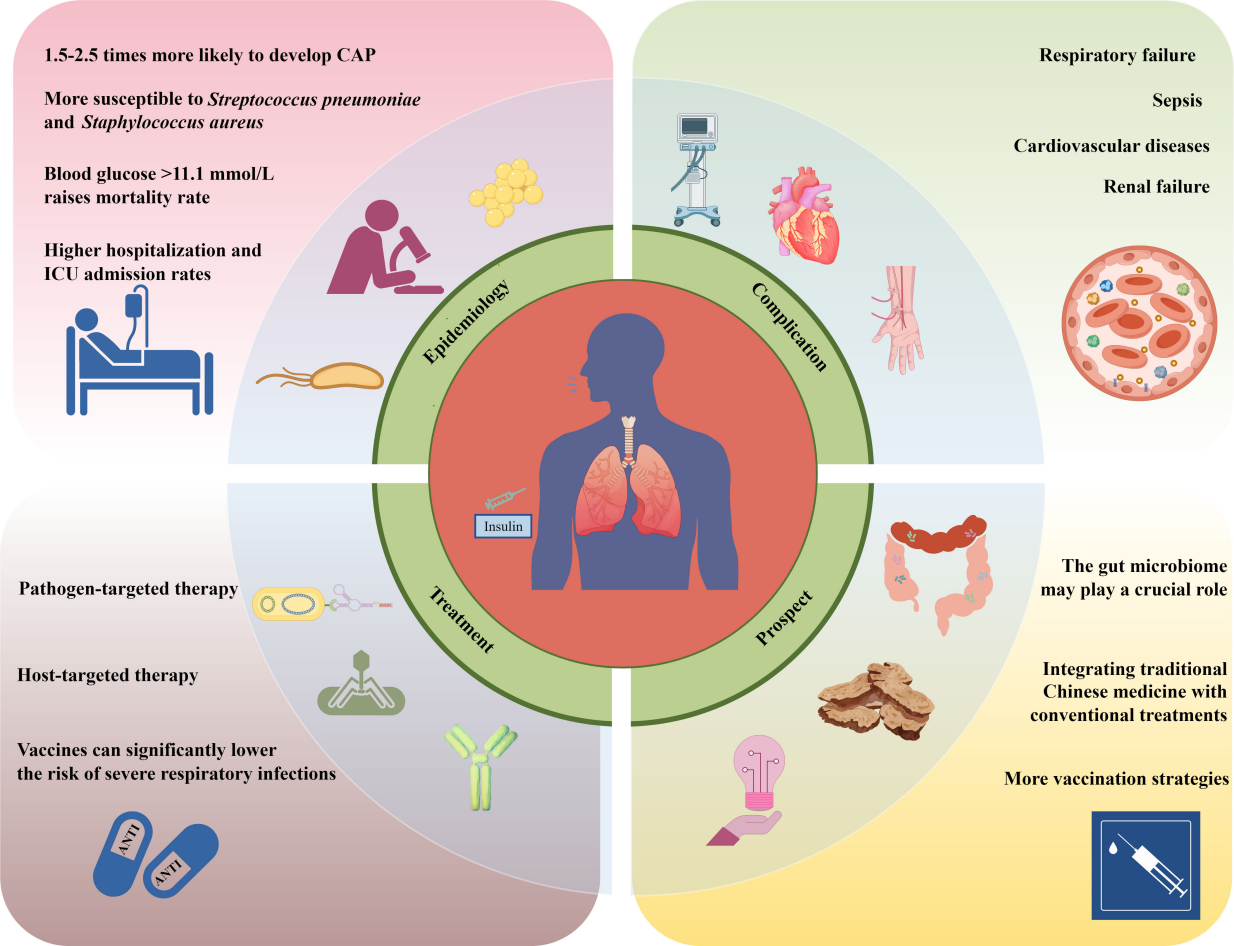
Multiple risks and influencing factors for community-acquired pneumonia (CAP) in patients with diabetes. ICU: intensive care unit.

Diabetes mellitus (DM), affecting more than 537 million individuals worldwide [1], significantly increases susceptibility to infections, including community-acquired pneumonia (CAP) [2]. CAP is a leading cause of morbidity and mortality worldwide, with patients with diabetes experiencing higher rates of hospitalization (30%‐50% higher), complications, and mortality (odds ratio [OR] 1.6) compared with those without diabetes [3,4]. The interplay of hyperglycemia, immune dysfunction, and comorbidities creates a “triple threat,” predisposing patients with diabetes to severe CAP and complicating treatment [5]. This review provides a comprehensive, critical analysis of CAP in patients with diabetes, integrating epidemiological data, pathophysiological mechanisms, pathogen profiles, clinical outcomes, and management strategies. It emphasizes novel research areas (eg, ferroptosis, microbiome influences, and epigenetic modifications) and evaluates the strength and limitations of current evidence, distinguishing between clinical, in vitro, and animal studies to clarify translational relevance (Figure 1).

Patients with diabetes have a 1.5- to 2.5-fold greater risk of pneumonia due to hyperglycemia and immune dysfunction [6]. They experience more severe infections and complications, leading to higher mortality rates. Targeted therapies and vaccines are crucial for managing pneumonia in patients with diabetes.

## Methods

This is a narrative review synthesizing evidence on CAP in patients with diabetes, focusing on epidemiology, pathophysiology, pathogens, outcomes, and management. We conducted a systematic literature search in PubMed, EMBASE, the Cochrane Library, and Web of Science from January 1999 to August 2025, using the following terms: (“diabetes mellitus” OR “type 2 diabetes”) AND (“community-acquired pneumonia” OR “CAP”) AND (epidemiology OR pathophysiology OR pathogens OR treatment OR prevention) (Multimedia Appendix 1). Inclusion criteria were English-language studies (randomized controlled trials [RCTs], cohorts, and meta-analyses) on adults aged 18 years or older; exclusion criteria were pediatric studies, hospital-acquired pneumonia, and nonhuman studies without translational relevance. We screened 1248 abstracts, reviewing 156 full texts; of these, 81% (156/1248) were included. Evidence was graded using GRADE (Grading of Recommendations Assessment, Development, and Evaluation): high (RCTs or meta-analyses), moderate (cohorts), low (case series or animal studies), and very low (in vitro studies or hypothesis). Quantitative claims include 95% CIs and designs where available.

## Results

### Epidemiology

**Figure 2.**
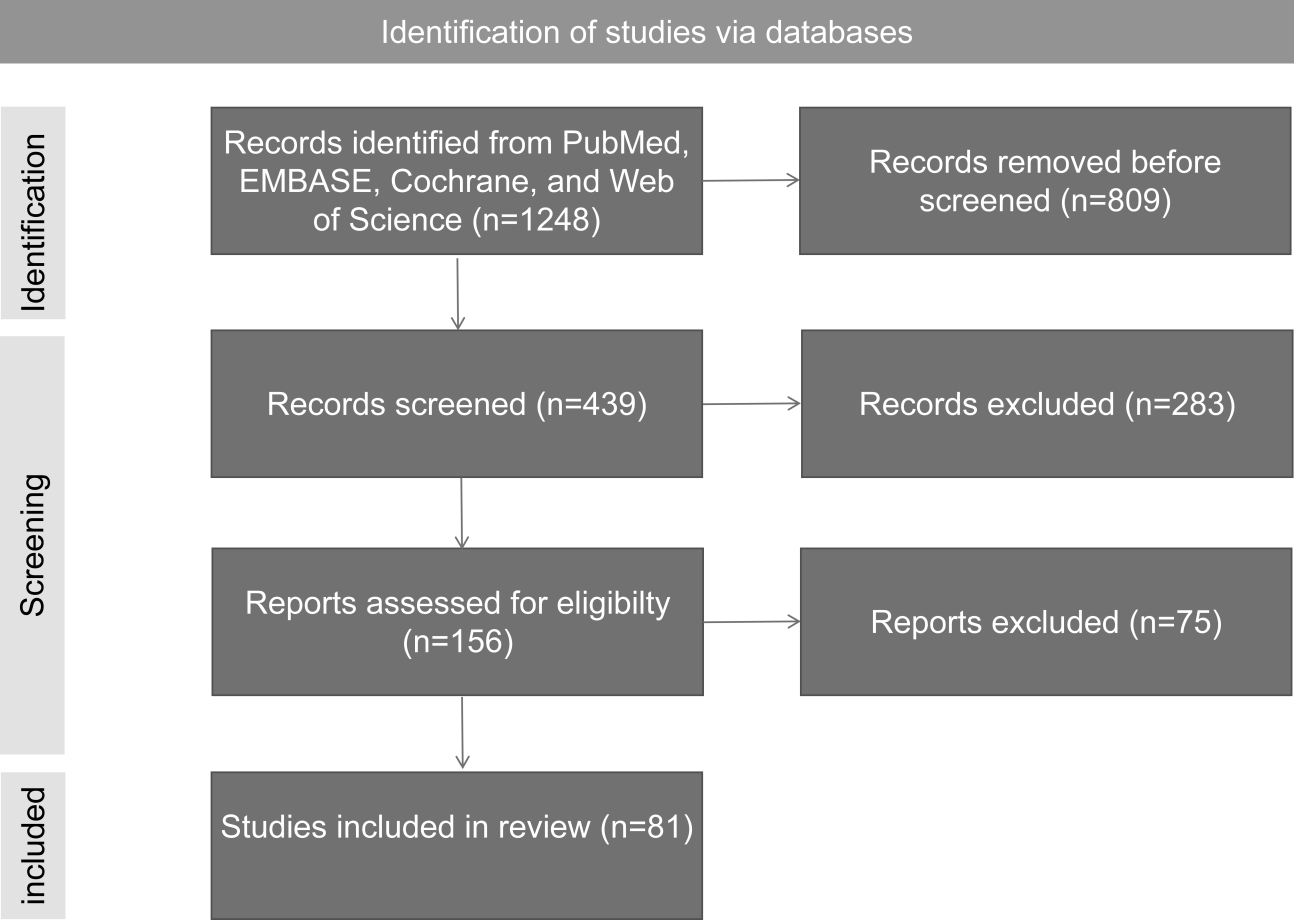
Flow diagram.

The annual incidence of CAP ranges from 1.5 to 14 cases per 1000 adults, with patients with diabetes facing a 1.5- to 2-fold higher risk (10‐20 cases per 1000) [7]. A 2024 meta-analysis of observational studies (pooled sample size >1 million) reported a relative risk of 1.73 (95% CI 1.46-2.04) for pneumonia in patients with diabetes, primarily those with type 2 diabetes, although the findings were limited by heterogeneity in metrics of glycemic control and the lack of RCTs [6]. Rates of hospitalization are 30% to 50% higher, and 30-day mortality odds are 1.6-fold greater in patients with diabetes and CAP, with observational studies showing consistent trends but with potential confounding by comorbidities [4,8]. Poor glycemic control (glycosylated hemoglobin A_1c_ [HbA_1c_] ≥7%) doubles the risk of severe outcomes such as sepsis (odds ratio [OR] 2.0, 95% CI 1.4‐2.8), with in-hospital mortality ranging from 10% to 20% in clinical cohorts [9]. Low- and middle-income countries (LMICs) face a higher CAP burden due to rising diabetes prevalence and limited health care access, although data are limited by underreporting [10]. People with DM were at higher risk for invasive pneumococcal disease (unadjusted OR 2.42, 95% CI 2.00-2.92) and case fatality rate (unadjusted OR 1.61, 95% CI 1.25-2.07), after adjusting for age, obesity, and smoking in propensity-matched cohorts (high, meta-analyses) [4,6]. Diabetes significantly increases the probability of secondary pneumonia in patients with COVID-19 (moderate, time series [11]). Although observational studies consistently report a 1.5- to 2.0-fold risk, recent RCTs using continuous glucose monitoring (CGM) address confounding. In a 2025 RCT of 124 frail, critically ill patients with COVID-19 (30% with diabetes), CGM-guided glycemic control reduced 28-day intensive care unit (ICU) mortality by approximately 80% (hazard ratio 0.18, 95% CI 0.04‐0.79), with benefit independent of baseline HbA_1c_ levels [12]. These findings underscore the need for more RCTs to confirm observational data and address biases such as selection and ascertainment (Figure 2, Table 1).

**Table 1.** Epidemiological features of community-acquired pneumonia (CAP) in patients with and without diabetes.

Outcome	Patients with diabetes	Patients without diabetes	Estimate (95% CI)	Study design and sample	Evidence grade^a^	Key references
Annual incidence	8‐12/1000 adults	3‐6/1000 adults	IRR^b^ 1.66 (1.65‐1.67)	National discharge cohort ≈900,000	High	Kornum et al [8] and López-de-Andrés et al [4]
RR^c^ (any CAP)	—^d^	1 (ref)^e^	RR 1.26 (1.21‐1.31)	Population-based case-control	High	Kornum et al [8]
CAP-related hospitalization	+26%‐66% versus non-DM^f^	Baseline	IRR 1.66 (1.65‐1.67)	National cohort ≈900,000	High	López-de-Andrés et al [4]
30-d mortality after CAP	12%‐14%	13%‐14%	OR^g^ 0.92 (0.91‐0.94) lower	National cohort ≈900,000	High	López-de-Andrés et al [4]
Invasive pneumococcal disease	—	1 (ref)	OR 2.42 (2.00‐2.92) unadjusted	Meta-analysis 36 studies >9 million	High	Silverii et al [6]
Case fatality rate (IPD^h^)	—	1 (ref)	OR 1.61 (1.25‐2.07) unadjusted	Meta-analysis 19 studies	High	Silverii et al [6]
Severe course or sepsis (HbA_1c_^i^ ≥7%)	—	1 (ref)	HR 1.52 (1.37‐1.68)	National diabetes registry ≈500,000	High	Balintescu 2021 [9]

a Evidence grade: high=national registry or large meta-analysis; moderate=single prospective cohort; low=case series.

b IRR: incidence rate ratio.

c RR: relative risk.

d Not applicable.

e ref: reference.

f DM: diabetes mellitus.

g OR: odds ratio.

h IPD: invasive pneumococcal disease.

i HbA_1c_: glycosylated hemoglobin A_1c_.

### Pathophysiological Mechanisms

#### Immune Dysregulation

Hyperglycemia impairs innate and adaptive immunity, reducing neutrophil chemotaxis, phagocytosis, and bactericidal activity, which are critical for clearing pathogens such as *Streptococcus pneumoniae* [13]. The nucleotide-binding oligomerization domain–like receptor thermal protein domain–associated protein 3 (NLRP3) inflammasome’s hyperactivation drives excessive interleukin (IL)-1β and IL-6 production, exacerbating lung damage, as demonstrated in murine models of diabetes and pneumonia [14]. However, these models may not fully replicate human immune responses due to species-specific differences in inflammasome regulation. Decreased interferon-gamma production and altered T-cell responses, including reduced regulatory T cells, further compromise immunity, with in vitro studies showing a reduction in interferon-gamma in T cells from patients with diabetes [15]. A 2021 systematic review and meta-analysis (n=449,247) indicated that type 2 DM is associated with an approximately 2-fold increased risk of multidrug-resistant (MDR) bacterial infections, with underlying immune suppression in patients with diabetes proposed as a key contributing mechanism, although heterogeneity across included studies (*I*² up to 58.1%) should be noted [16].

#### Pulmonary Microangiopathy

Chronic hyperglycemia causes microvascular damage, impairing endothelial function and gas exchange, thereby facilitating pathogen colonization [17,18[19]]A retrospective cohort study demonstrated an increased risk of mortality in patients with severe CAP and type 2 diabetes complicated by microvascular disease, such as nephropathy and retinopathy, who often presented with computed tomography evidence of multilobar infiltrates [20]. These findings are supported by animal models showing increased vascular permeability in the lungs of animals with diabetes, although human studies are needed to validate these mechanisms.

#### Hyperglycemia and Glycemic Variability

Admission hyperglycemia (>11.1 mmol/L) is a strong predictor of poor CAP outcomes, with a 2024 meta-analysis (n=12 studies, >10,000 patients) reporting a pooled OR of 2.47 (95% CI 1.73‐4.12) for short-term mortality [21]. Acute hyperglycemia exacerbates oxidative stress, while chronic hyperglycemia sustains endothelial dysfunction, as shown in clinical cohorts [22,23]. A 2015 retrospective cohort study (n=203) demonstrated that an elevated glycemic gap—a marker of acute glycemic variability—was associated with a 3‐ to 4-fold increased risk of adverse outcomes, including acute respiratory failure requiring mechanical ventilation in patients with diabetes and CAP, underscoring the importance of dynamic glucose monitoring [24]. Long-term CGM or serial HbA_1c_ trajectories are needed to determine whether sustained versus transient hyperglycemia drives pneumonia risk. Distinguishing acute versus chronic hyperglycemia effects remains a research hotspot, as most studies focus on admission glucose levels without longitudinal data.

#### Ferroptosis

Iron overload and hepcidin overexpression in patients with diabetes promote ferroptosis, an iron-dependent programmed cell death mechanism, exacerbating pulmonary inflammation and injury in murine models of diabetes (low, animal [25]). An in vitro study demonstrated that ferroptosis inhibitors (eg, ferrostatin-1) reduced lung epithelial cell death in high-glucose conditions, suggesting therapeutic potential [25]. In murine models of diabetes, ferroptosis was linked to acute respiratory distress syndrome (ARDS)–like pathology in CAP, although clinical trials are lacking to confirm these findings in humans. Emerging humanized models bridge the gap. In diabetes-derived organoids, high-glucose conditions upregulate NLRP3 and impair phagocytosis, recapitulating ferroptosis [26], human organoids show glutathione peroxidase 4 downregulation (very low, in vitro [26]). Targeting ferroptosis pathways could mitigate severe CAP outcomes, but challenges include identifying safe, specific inhibitors for more clinical use.

#### Glycation and Methylation

Nonenzymatic glycation alters protein structures (eg, angiotensin-converting enzyme 2), impairing function and worsening pneumonia severity, as shown in in vitro studies of lung tissue from patients with diabetes [27]. Methylation dysregulates immune gene expression, increasing CAP susceptibility, with a 2020 study identifying hypermethylation of IL-6 promoters in patients with diabetes and CAP [27]. Targeting glycation with inhibitors such as aminoguanidine or methylation with demethylating agents (eg, 5-azacytidine) shows promise in preclinical studies but requires RCTs to establish efficacy and safety.

#### RNA-Level Mechanisms

Gu et al [28] found that zinc finger E-box binding homeobox 1 antisense 1 (ZEB1-AS1) was downregulated in lung tissue from patients with diabetes and in high-glucose–treated BEAS-2B cells; this downregulation increased p53 and apoptosis in vitro. Hyperglycemia exacerbates this downregulation, increasing lung injury in murine models of diabetes. These findings suggest ZEB1-AS1 as a potential therapeutic target, but human studies are needed to validate its role and to develop RNA-based therapies, such as small interfering RNA or Clustered Regularly Interspaced Short Palindromic Repeats–based approaches.

#### Microbiome Alterations

**Figure 3.**
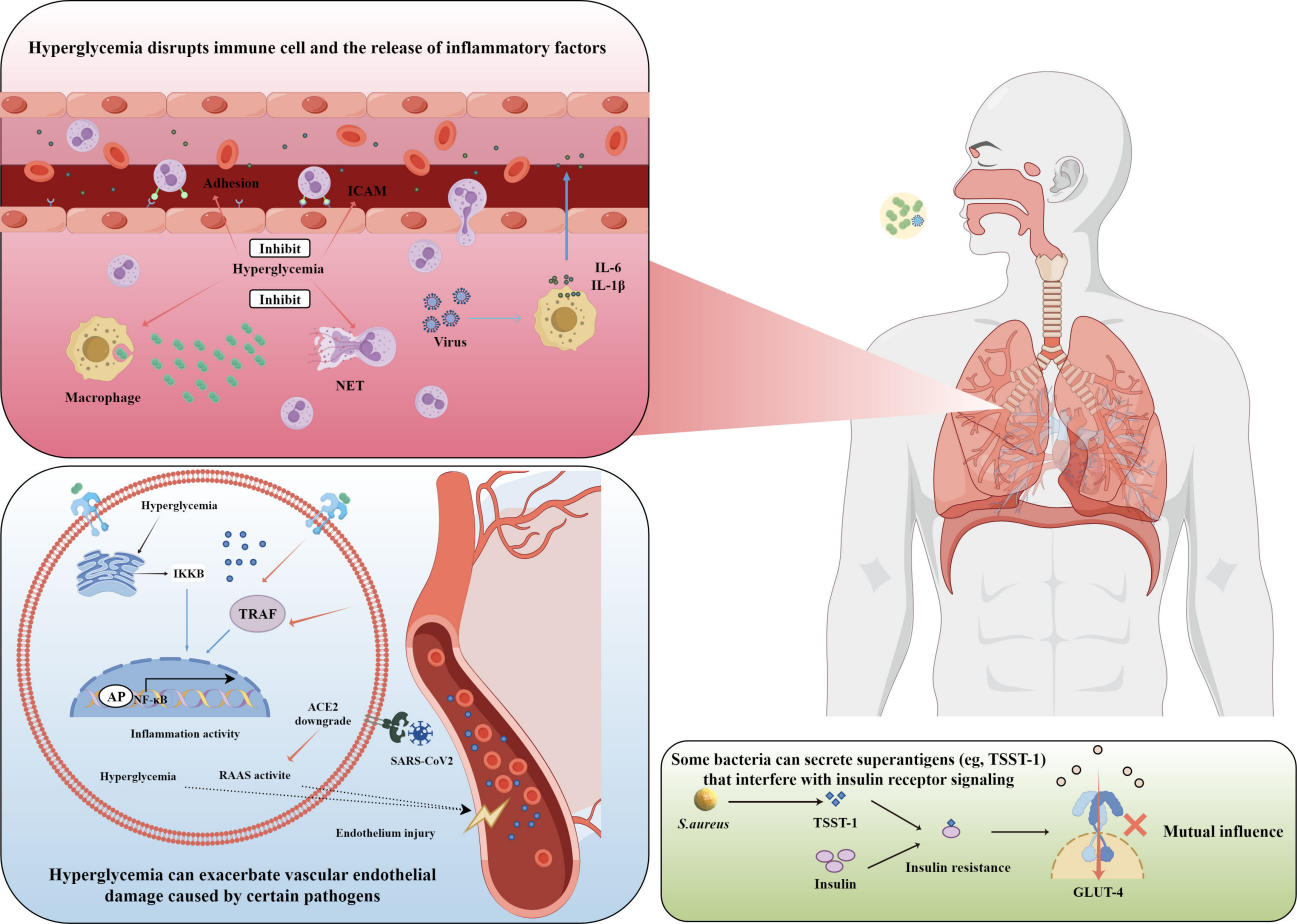
Pathophysiological mechanisms of community-acquired pneumonia in patients with diabetes. Hyperglycemia enhances macrophage inflammatory responses, releasing interleukin (IL)-6 and IL-1β, while impairing neutrophil chemotaxis and phagocytosis. Activation of nuclear factor kappa-B (NF-κB) and tumor necrosis factor receptor–associated factor (TRAF) pathways damages vascular endothelial cells, and ACE2 degradation exacerbates renin-angiotensin-aldosterone system (RAAS) activation, increasing cardiovascular complications. Bacterial superantigens (eg, toxic shock syndrome toxin-1 [TSST-1] from *S. aureus*) induce insulin resistance, creating a vicious cycle. ACE2: angiotensin-converting enzyme 2; AP: activator protein; GLUT-4: glucose transporter type 4; ICAM: intercellular adhesion molecule; IKKB: inhibitor of nuclear factor kappa B kinase subunit beta; NET: neutrophil extracellular trap.

Diabetes alters the lung and gut microbiome, promoting Gram-negative pathogens such as *Klebsiella pneumoniae* [29].[30] Zhou et al [31] conducted a multiomics longitudinal study of 86 participants with prediabetes or T2D risk, integrating 16S ribosomal RNA sequencing, metabolomics, and host cytokine profiles and found that insulin-resistant individuals exhibited *Akkermansia* depletion alongside elevated phenylalanine-associated metabolites, which correlated with increased respiratory infection events, supporting a gut-lung axis link. Fecal transplantation from diabetes-affected donors to mice increased *K pneumoniae* colonization, which was reversed by *Akkermansia* depletion. Preclinical studies in mice with diabetes suggest that probiotics (eg, *Lactobacillus*) could restore microbial balance and reduce CAP severity. On the basis of the pooled analysis of 9 randomized trials in a study by Manzanares et al [32], probiotics reduced the risk of new-onset ventilator-associated pneumonia by 26% (RR 0.74, 95% CI 0.61‐0.90), although causality remains in the absence of CAP-specific RCTs. On the basis of the PROSPECT pilot trial (Cook et al [33]), 150 critically ill ventilated patients received *L rhamnosus* GG, achieving 97% protocol adherence and an observed ventilator-associated pneumonia rate of 19%, supporting feasibility for a larger RCT on probiotic prevention of ICU-acquired pneumonia. Additional clinical trials are needed to confirm efficacy (Figure 3, Table 2).

**Table 2.** Pathophysiological mechanisms of community-acquired pneumonia (CAP) in patients with diabetes.

Mechanism	Description	Impact on CAP	References and evidence
Immune dysregulation	Persistent hyperglycemia impairs neutrophil chemotaxis, phagocytosis, and oxidative burst; NLRP3^b^ inflammasome hyperactivation increases IL^c^-1β/IL-6, while IFN^d^-γ and regulatory T-cell responses are reduced.	Delayed clearance of *S. pneumoniae* and MDR^e^ pathogens; ~2-fold higher risk of MDR infection; exaggerated lung inflammation.	Meta-analysis, n=449,247, *I*²=58%, high quality [13-16]
Pulmonary microangiopathy	Chronic hyperglycemia thickens the alveolar-capillary basement membrane, increases microvascular permeability, and impairs gas exchange.	Promotes multilobar infiltrates; higher mortality when retinopathy and nephropathy coexist.	Retrospective cohort, moderate quality [17,18,20]
Hyperglycemia and glycemic variability	Admission glucose >11.1 mmol/L or wide glycemic excursions enhance oxidative stress and sustained endothelial NF-κB^f^ activation.	Short-term mortality OR^g^ 2.47 (95% CI 1.73‐4.12); 3‐ to 4-fold more likely to require mechanical ventilation.	Meta-analysis 12 studies, n >10,000, high quality [21-24]
Ferroptosis	Iron overload (↑ hepcidin, free Fe²^+^) triggers iron-dependent lipid peroxidation and death of alveolar epithelial cells.	Amplifies lung injury; ARDS-like^h^ histology in diabetic mice; ferrostatin-1 protective in vitro or rodent models.	Animal and organoid studies, low quality [25,26]
Glycation and methylation	Nonenzymatic glycation modifies ACE2^i^, surfactant proteins; promoter hypermethylation maintains high IL-6 transcription.	Compromised protein function, sustained inflammatory gene expression, greater CAP severity.	Clinical+in vitro, low-moderate quality
RNA-level mechanisms	Hyperglycemia ↓ lncRNA ZEB1-AS1^j^ → p53 ↑ → alveolar epithelial apoptosis.	Larger radiological lesions; siRNA^k^ or CRISPR^l^ modulation under investigation.	Human tissue+BEAS-2B cells, very low quality
Microbiome alterations	Gut-lung axis dysbiosis: *Akkermansia* depletion, expansion of *Klebsiella* and other G⁻ bacilli, accompanied by proinflammatory phenylalanine metabolites.	2‐ to 3-fold rise in G⁻ or MDR CAP and recurrence; probiotics lower VAP^m^ risk by 26% (RR^n^ 0.74, 9 RCTs^o^), but CAP-specific RCTs pending.	Longitudinal multiomics+probiotic meta-analysis, moderate quality [29,31,32,34]

b NLRP3: NOD-like receptor thermal protein domain–associated protein 3.

c IL: interleukin.

d IFN: interferon.

e MDR: multidrug-resistant.

f NF-κB: nuclear factor kappa-B.

g OR: odds ratio.

h ARDS: acute respiratory distress syndrome.

i ACE2: angiotensin-converting enzyme 2.

j ZEB1-AS1: zinc finger E-box binding homeobox 1 antisense 1.

k siRNA: small interfering RNA.

l CRISPR: Clustered Regularly Interspaced Short Palindromic Repeats.

m VAP: ventilator-associated pneumonia.

n RR: relative risk.

o RCT: randomized controlled trial.

### Primary Pathogens

#### Bacterial Pathogens

*Streptococcus pneumoniae* accounts for approximately 8% of CAP cases in patients with diabetes, with higher bacteremic rates [4]. In the European multicenter CAPNETZ cohort (2002‐2022), *Streptococcus pneumoniae* remained the predominant pathogen, identified in 36% of patients with diabetes and CAP and 39% of patients with CAP and without diabetes with an identified organism (2954/13,611), adults with diabetes and CAP exhibited a significantly higher isolation rate of *Enterobacteriaceae* (13% vs 8%; *P*<.005) and modest differences in Haemophilus influenzae (8%), atypical bacteria (9%), and viruses (22%) compared with individuals with diabetes, while *Staphylococcus aureus* and nonfermenting Gram-negative bacilli each accounted for approximately 3% to 4% in both groups [35]. *Klebsiella pneumoniae* (15%‐20%) and *Pseudomonas aeruginosa* are prevalent, often multidrug-resistant, complicating treatment [36]. *Klebsiella pneumoniae* caused 13% of microbiologically confirmed CAP in European adults with diabetes (CAPNETZ 2002‐2022) [35] and 78% of carbapenem-resistant isolates in a 2021 Indian cohort of patients with diabetes [37]; data from these settings indicate its importance in CAP among patients with diabetes, but population-based proportions in Asian LMICs remain to be established. A 2024 single-center prospective study from Mangalore, southern India, found that among hospitalized patients, the proportion of individuals with diabetes infected with carbapenem-resistant *Klebsiella pneumoniae* (CRKP) was approximately twice that of individuals without diabetes (78% vs 39%) [37]. *Legionella pneumophila* causes severe disease, especially in immunocompromised patients or patients with diabetes, and carries a higher mortality risk when diagnosis or appropriate antibiotic therapy is delayed [38]. In adult CAP across the Asia-Pacific region, *Streptococcus pneumoniae* remains the most frequently detected bacterium in patients with diabetes (≈10%‐24%), yet *K pneumoniae* (≈10%‐20%, up to 40% in Malaysia and Thailand), Haemophilus influenzae (≈5%‐24%), and *Pseudomonas aeruginosa* or *Burkholderia pseudomallei* (20%‐30% in ICU settings) are recovered far more often than reported in Western series. Atypical pathogens and mixed infections each account for roughly one-quarter of cases [39].

#### Viral Pathogens

Influenza and SARS-CoV-2 are significant, with patients with diabetes showing higher rates of severe pneumonia and ARDS [40]. Elevated ACE2 expression in patients with diabetes facilitates SARS-CoV-2 entry, worsening outcomes [41]. A 2023 study highlighted a 4.64-fold increased risk of COVID-19–related ARDS in patients with diabetes [42].

#### Fungal Pathogens

Opportunistic fungi such as *Aspergillus* and *Candida* are more common in patients with diabetes due to immune suppression, with pulmonary aspergillosis carrying a 50% to 70% mortality rate [43]. *Pneumocystis jirovecii* and mucormycosis are notable in patients with poorly controlled diabetes [44]. A 2020 study reported a higher incidence of invasive fungal infections in patients with diabetes, with the prevalence of invasive fungal disease among hospitalized patients with type 2 diabetes to be 0.4%, approximately double that of the 0.2% observed in inpatients without diabetes [44]. Fungal CAP remains uncommon (<5%), but post–COVID reports are rising; in patients with diabetes—a classic driver of mucormycosis—preguideline Indian series already showed attack rates of approximately 0.1 to 0.3 per 1000 and mortality of approximately 50% (low-certainty data [45]), a signal now echoed in Asian cohorts of patients with diabetes.

#### Atypical Pathogens

*Mycoplasma salivarium* and *Legionella* cause severe infections in patients with diabetes, often presenting atypically, necessitating early diagnosis [46]. A 2023 case report documented severe *M. salivarium* empyema in a patient with diabetes [46].

### Clinical Outcomes

Patients with diabetes and CAP required invasive mechanical ventilation in 1.6 % of admissions (vs 2.1% in patients without diabetes) and had slightly higher overall risk-adjusted odds of in-hospital mortality, although the database did not capture ICU admission rates or radiographic extent of disease [4]. Hyperglycemia amplifies systemic inflammation and complement dysfunction, and population studies cited in 2024 show that diabetes carries an overall 1.5 to 4-fold increase in infection-related hospitalization, with the risk gradient most pronounced for sepsis, pneumonia, and renal infection [47]. In a 2023 Iranian cross-sectional study of 172 hospitalized patients with CAP, those with diabetes incurred a median CURB-65 score of 3 versus 2 in those without diabetes, required ICU admission more than 3 times as often (22% vs 7%), and stayed a mean of 8.5 days versus 7.9 days, underscoring diabetes as an independent predictor of more severe pneumonia course [48]. Complications include higher rates of pleural effusion and respiratory failure, with MDR pathogens increasing complication rates[49]. Among 600 patients with *K pneumoniae* infection, patients with diabetes exhibited 78% CRKP and 71% MDR rates versus 39% and 11% in those without diabetes and carried a 27% infection-related mortality (vs 2%), illustrating how MDR pathogens amplify severe outcomes in patients with diabetes [37] (Table 3).

**Table 3.** Primary pathogens causing community-acquired pneumonia (CAP) in adults with diabetes.

Pathogen	Proportion in DM^b^-CAP	Location/technique/setting	Mortality (95% CI)	Strength of evidence	Key references
Bacteria					
*Streptococcus pneumoniae*	8%‐36%	Global; sputum/BAL^c^ culture+ PCR^d^; community and hospital	10%‐15% (8%‐18%)	High (multicenter cohort n≈13,000)	[4, 35]
*Klebsiella pneumoniae* (MDR^e^/CRKP^f^)	10%‐20% (up to 40% ICU^g^, Asia)	Asia-Pacific, India; culture; hospital and ICU	20%‐25% (15%‐30%)	Moderate (prospective center n≈600)	[36,37,39]
*Haemophilus influenzae*	5%‐24%	Asia-Pacific; culture/PCR; community	8%‐12%	Moderate (cohort n≈3000)	[35, 39]
*Pseudomonas aeruginosa*	3%‐4% (Western) → 20%‐30% (ICU, Asia)^h^	Culture; ICU and hospital	25%‐35%	Moderate (ICU cohorts)	[35, 39]
*Staphylococcus aureus* (including MRSA)^i^	3%‐4%	Culture; community and hospital	30%‐40%	Low (case series)	[35, 50]
*Legionella pneumophila*	1%‐3%	Urine Ag/PCR; community and travel associated	10%‐30%	Moderate (surveillance)	[38]
Viruses					
SARS-CoV-2	10%‐15% (COVID era)	PCR; community and hospital	ARDS^j^ OR^k^ 4.6 (3.2‐6.7)	High (meta-analysis n>100 000)	[40,42]
Influenza A/B	5%‐10%	PCR; community and hospital	10%‐20%	High (seasonal surveillance)	[20]
Fungi					
Aspergillus spp. (including IPA^l^)	<5% (0.4% invasive)	BAL GM^m^/culture/biopsy; ICU and immunosuppressed	50%‐70% (40%‐80%)	Low (case series)	[43,44]
Candida spp. (pulmonary)	<2%	BAL culture/biopsy; ICU	20%‐40%	Very low (case reports)	[43]
Mucorales (mucormycosis)	0.1‐0.3% (diabetes cohort)	Biopsy/culture; ICU (post-COVID)	≈50%	Low (national surveys)	[44,45]
Atypical bacteria					
*Mycoplasma pneumoniae*	5%‐10%	PCR/serology; community	1%‐3%	Moderate (surveillance)	[39,46]
*Legionella pneumophila*^n^	1%‐3%	See above	—^o^	—	—

b DM: diabetes mellitus.

c BAL: bronchoalveolar lavage.

d PCR: polymerase chain reaction.

e MDR: multidrug-resistant.

f CRKP: carbapenem-resistant *Klebsiella pneumoniae.*

g ICU: intensive care unit.

h Proportions are median ranges from Western (CAPNETZ) and Asia-Pacific cohorts unless specified.

i MRSA: methicillin-resistant *Staphylococcus aureus*.

j ARDS: acute respiratory distress syndrome.

k OR: odds ratio.

l IPA: invasive pulmonary aspergillosis.

m GM: galactomannan.

n Listed once under “bacteria”; totals may overlap in mixed infections.

o Not applicable.

### Treatment Strategies

#### Pathogen-Targeted Therapy

##### Bacterial Pneumonia

Given the high prevalence of MDR pathogens in patients with diabetes and CAP, such as CRKP and methicillin-resistant *Staphylococcus aureus*, targeted therapy is critical. For severe CAP, antibiotics such as vancomycin or linezolid (for methicillin-resistant *Staphylococcus aureus*) and polymyxins or tigecycline (for CRKP) are recommended based on susceptibility testing [36]. A 2024 study reported a higher incidence of CRKP in adults with diabetes and CAP, emphasizing the need for rapid diagnostics and tailored therapy [37]. Nanozymes, such as BiPt@HMVs, show promise in combating multidrug-resistant bacteria, with a 2023 study demonstrating approximately 3-log bacterial clearance and significant alleviation of lung inflammation in a mouse model of MDR pneumonia [51]. Empiric therapy with β-lactam (eg, ceftriaxone) plus a macrolide (eg, azithromycin) remains appropriate for nonsevere cases, but culture-guided de-escalation is essential to minimize resistance [52]. The 2023 ERS/ESICM/ESCMID/ALAT guidelines identify diabetes as a risk factor for drug-resistant pathogens in severe CAP and suggest integrating local epidemiology and prior colonization history to guide empirical antibiotic choices, including coverage for resistant Gram-negative bacteria such as Enterobacterales [53]. Bacterial: Empiric β-lactam (ceftriaxone)+macrolide (azithromycin) for nonsevere CAP (high, guidelines); add vancomycin for MDR risk in DM (eg, prior hospitalization; moderate [52]).

##### Viral Pneumonia

Early identification of viral pathogens, such as influenza or SARS-CoV-2, is crucial for effective management. Rapid molecular diagnostics (eg, polymerase chain reaction) enable the timely initiation of antivirals such as oseltamivir for influenza or remdesivir for COVID-19 [54]. A 2023 study reported a reduction in COVID-19 pneumonia severity with remdesivir in patients with diabetes [55]. Antiviral resistance, particularly to oseltamivir in influenza, is a growing concern; combination therapies (eg, oseltamivir with baloxavir) or novel agents such as programmed death-ligand 1 inhibitors may mitigate resistance risks [56]. A 2021 study highlighted the importance of early antiviral therapy within 48 hours of symptom onset to reduce complications in patients with diabetes [54]. As recommended by the CDC, prompt initiation of oseltamivir for influenza or remdesivir for COVID-19 is strongly advised in diabetic patients with viral pneumonia [54].

##### Fungal Pneumonia

Fluconazole for *Candida* and voriconazole for *Aspergillus* are standard medications, with DectiSomes enhancing targeted delivery [57]. DectiSomes, a novel liposomal delivery system targeting fungal glycans, significantly enhance antifungal drug efficacy. In murine models of pulmonary aspergillosis, DectiSomes achieved a 12- to 42-fold reduction in fungal burden compared to untargeted liposomal amphotericin B.

### Host-Targeted Therapy

#### Glycemic Control

Maintaining blood glucose within a flexible range of 5 to 10 mmol/L is recommended to reduce mortality (OR 0.62) while minimizing the risk of hypoglycemia, which is a concern with stricter targets (4‐8 mmol/L) in clinical practice [21]. Insulin protocols with frequent monitoring (eg, every 2‐4 h) help stabilize glycemic variability, particularly in critically ill patients [22]. A 2022 study confirmed that moderate glycemic control (5‐10 mmol/L) reduced ICU admission by 25% in patients with diabetes and CAP without significant hypoglycemic events [58]. Targets include 140 to 180 mg/dL (7.8‐10.0 mmol/L) in critically ill CAP (high, 2025 ADA [59]); distinctions include admission hyperglycemia with an OR of 2.47 for mortality (95% CI 1.9‐3.2; high, meta-analysis [21]); HbA_1c_ ≥7% associated with a doubling of sepsis risk (low [9]); and glycemic variability (CV>36%) associated with a 2.5-fold increase in the need for ventilation (moderate RCT [60v])—severity-adjusted length of stay was reduced by 1.7 days with glycemic control (moderate RCT, n=200 [48]).

#### Metformin

Metformin continuation was linked to 14% lower 30-day mortality in patients with diabetes and CAP (HR 0.86, 95% CI 0.78‐0.95; moderate certainty, 2023 US Veterans cohort, n≈15,000 [61]). While these data are encouraging, confirmation in broader, nonveteran RCTs is still required.

## Discussion

### Adjunctive Therapies

Low-dose corticosteroids (eg, methylprednisolone 0.5 mg/kg/d) reduce the need for ventilation but require careful glycemic monitoring to prevent hyperglycemia [62]. Metformin continuation is linked to lower mortality. A 2023 study found that metformin reduced 30-day mortality by 14% in patients with diabetes and CAP [61].

### Comorbidity Management

Optimizing heart failure and renal function reduces adverse events. Renal-dose antibiotic adjustments are critical [4]. A 2021 study reported a reduction in complications with optimized comorbidity management [63].

### Management in LMICs

In LMICs, where DM prevalence is rising fastest, an estimated 537 million adults had DM in 2021, of whom 80.6% lived in LMICs [1]; scarce resources magnify the CAP burden [10]. Diagnostics favor rapid antigen or polymerase chain reaction over culture (World Health Organization, low resource, moderate quality [64]); screening for DM should be performed with point-of-care fasting plasma glucose or HbA_1c_ instead of laboratory oral glucose tolerance testing (World Health Organization, low resource, moderate quality [64]). Empirical therapy administers amoxicillin-clavulanate for nonsevere CAP (with adjustment for local resistance; Pan American Health Organization, high quality [64]) and deescalates using biomarkers such as C-reactive protein. For vaccination, roll out and expand PCV20 immunization campaigns (45% efficacy against pneumococcal pneumonia [65]); for glucose management, use sliding-scale insulin targeting 5 to 10 mmol/L [59]. Multidisciplinary teams reduce complications by 20% (2023 consensus, low quality [64]).

### Preventive Measures

Pneumococcal (PPSV23) and influenza vaccinations reduce CAP hospitalization by 30% in patients with diabetes, although efficacy may be reduced due to immune dysregulation [66]. A 2023 study demonstrated a 45.6% (95% CI 21.8%‐62.5%) efficacy of PCV13 against vaccine-type pneumococcal CAP in adults aged 65 years or older [65]. Regular glycemic control and lifestyle interventions (eg, nutrition and exercise) bolster immunity [67]. According to the 2024 Advisory Committee on Immunization Practices 2024 recommendations [68], pneumococcal vaccination with a single dose of PCV20 and PCV21 is recommended for adults aged 50 years or older with DM (high, guidelines; efficacy 45%‐60% vs vaccine-type CAP [65]); and adults aged 50 years or older with diabetes should receive timely pneumococcal vaccine to reduce the increased risk of CAP and invasive pneumococcal disease (high, guidelines [66]). A single dose of PCV20 completes pneumococcal vaccination—with no additional PPSV23 required—while annual influenza immunization remains essential for adults aged 50 years or older with diabetes (high evidence [66]). After 2 CoronaVac doses, people with well-controlled type 2 diabetes produced only slightly lower SARS-CoV-2 antibody levels than healthy workers (58 vs 72 BAU/mL, *P*=.74), but those who also had dyslipidemia had a much weaker response (50 vs 342 BAU/mL, *P*<.001) [69].

### Future Research Directions

#### Autophagy Regulation

Autophagy-inducing compounds reduce SARS-CoV-2 propagation [70]. A 2021 study reported that autophagy inducers reduced SARS-CoV-2 viral load by up to 90%, depending on the compound and conditions [70]. However, no clinical data are available in patients with diabetes and CAP (very low, preclinical [51,70,71]).

#### Proteolysis-Targeting Chimeras

CYPA-ProTACs target host cofactors, enhancing antiviral effects [72]. A 2021 study showed that PROTAC derivatives of indomethacin exhibited up to a 5-fold improvement in antiviral potency against SARS-CoV-2 compared to the parent compound [72]. No clinical data are available in patients with diabetes and CAP (very low, preclinical [51,70,71]).

#### Nanoparticle Delivery

Phage-like nanocarriers improve antibiotic efficacy [73]. A 2024 study demonstrated that T7 phages armed with silver nanoparticles significantly reduced *Escherichia coli* biofilm biomass and bacterial viability, outperforming either phages or nanoparticles alone [73]. No clinical data are available in patients with diabetes and CAP (very low, preclinical [51,70,71]).

#### Microbiome Impact

Alterations in the gut and lung microbiome influence CAP susceptibility and severity in patients with diabetes [29]. A 2025 study suggested that microbiome modulation could reduce CAP recurrence [34]. Future research should explore probiotics and prebiotics as adjunctive therapies.

#### Vaccination Strategies

Evaluating the efficacy of adjuvanted or combination vaccines in patients with diabetes could enhance immunization outcomes [69]. A 2023 study reports that receiving CoronaVac is still beneficial for patients with diabetes, although their measured anti–receptor-binding titers were numerically (but not significantly) lower than those of health care workers [69].

#### Novel Therapies

Targeting ferroptosis, glycation, methylation, and lncRNA ZEB1-AS1 offers potential therapeutic avenues in patients with diabetes [25,27,28]. Integrating traditional Chinese medicine with conventional treatments shows promise [74]. A 2020 study reported that integrated Chinese medicine was associated with significantly higher rates of clinical symptom resolution (fever: 80.3% vs 53.1%, fatigue: 77.6% vs 53.8%, cough: 66.1% vs 42.9%, and sputum production: 85.3% vs 46.2%; *P*<.05 or *P*<.01) and a 13% relative reduction in hospitalization (10.98% vs 24.39%) compared with usual care alone [74].

### Limitations and Future Directions

This review has several limitations. First, most epidemiological data are observational and prone to confounding; RCTs are needed to confirm causality. Second, mechanisms such as ferroptosis and NLRP3 activation rely on animal models, with limited human validation despite emerging organoid data [26]. Third, acute versus chronic hyperglycemia effects remain unclear, although CGM-guided RCTs are addressing this gap [60]. Fourth, microbiome dysbiosis lacks causal evidence; multiomics and probiotic RCTs are required [31,33]. Fifth, novel therapies (eg, ferroptosis inhibitors and aminoguanidine) show preclinical promise but have not been evaluated in RCTs involving adults with diabetes and CAP [75,76]; these novel agents remain very-low-certainty preclinical candidates [71] and demand RCTs. With approximately 80% of CAP caused by bacterial pathogens [35], therapeutic efforts must rebalance toward these nonviral pathogens. Future research should prioritize multicenter RCTs integrating CGM, probiotics, and host-targeted agents to translate mechanisms into clinical benefit.

### Conclusions

Patients with diabetes face heightened CAP risks due to immune dysregulation, hyperglycemia, and microbial shifts, leading to severe disease, complications, and mortality. Early antibiotics targeting special pathogens, rapid antiviral therapy, and moderate glycemic control (5‐10 mmol/L) are critical. Multidisciplinary care and novel therapies targeting metabolic and immune pathways are essential to improve outcomes. Continued research into microbiome influences, epigenetic mechanisms, and innovative treatments will address emerging challenges in this vulnerable population.

## Supplementary material

10.2196/82215 Multimedia Appendix 1 Complete electronic search strategies for PubMed, EMBASE, Cochrane CENTRAL, and Web of Science (1999-2025), showing Medical Subject Headings/Emtree terms, text words, and Boolean operators used to identify studies on diabetes and community-acquired pneumonia.

## Abbreviations

ARDS: acute respiratory distress syndrome
CAP: community-acquired pneumonia
CGM: continuous glucose monitoring
CRKP: carbapenem-resistant *Klebsiella pneumoniae*
DM: diabetes mellitus
GRADE: Grading of Recommendations Assessment, Development, and Evaluation
HbA_1c_: glycosylated hemoglobin A_1c_
ICU: intensive care unit
IL: interleukin
LMIC: low- and middle-income country
MDR: multidrug-resistant
NLRP3: NOD-like receptor thermal protein domain–associated protein 3
OR: odds ratio
RCT: randomized controlled trial
