# Supplementary material for: Community-Acquired Pneumonia in Patients With Diabetes: Narrative Review
Source: JMIR Diabetes. 2026 Mar 10;11:e82215. doi: 10.2196/82215 (PMC12975005; doi:10.2196/82215)
Supplement: Multimedia Appendix 1 [file diabetes-v11-e82215-s001.docx]

**Supplementary Material 1. Complete electronic search strategies**

Database: PubMed

Date of last search: 31 August 2025

Time span: 1999/01/01–2025/08/31

Search string:

("diabetes mellitus"[MeSH] OR "diabetes mellitus, type 2"[MeSH] OR diabetes[tiab] OR diabetic[tiab]) AND ("community-acquired pneumonia"[MeSH] OR "pneumonia, bacterial"[MeSH] OR "pneumonia, viral"[MeSH] OR community-acquired[tiab] OR CAP[tiab]) AND (epidemiology[tiab] OR incidence[tiab] OR prevalence[tiab] OR mortality[tiab] OR pathophysiology[tiab] OR etiology[tiab] OR microbiology[tiab] OR pathogen*[tiab] OR treatment[tiab] OR therapy[tiab] OR prevention[tiab] OR vaccine*[tiab]) NOT (hospital-acquired[tiab] OR nosocomial[tiab] OR "healthcare-associated"[tiab]) Limits: Humans, English, Adult (≥19 y)

Database: EMBASE

Time span: 1999–2025 Week 35

EMTREE terms:

('diabetes mellitus'/exp OR 'type 2 diabetes mellitus'/exp OR diabetes:ti,ab OR diabetic:ti,ab) AND ('community acquired pneumonia'/exp OR 'bacterial pneumonia'/exp OR 'viral pneumonia'/exp OR 'community acquired':ti,ab OR CAP:ti,ab) AND (epidemiology:ti,ab OR incidence:ti,ab OR prevalence:ti,ab OR mortality:ti,ab OR pathophysiology:ti,ab OR etiology:ti,ab OR microbiology:ti,ab OR pathogen*:ti,ab OR treatment:ti,ab OR therapy:ti,ab OR prevention:ti,ab OR vaccine*:ti,ab) NOT (hospital acquired:ti,ab OR nosocomial:ti,ab OR healthcare associated:ti,ab) AND [humans]/lim AND [adult]/lim AND [english]/lim

Database: Cochrane Library (CENTRAL)

#1 MeSH descriptor: [Diabetes Mellitus] explode all trees

#2 MeSH descriptor: [Diabetes Mellitus, Type 2] explode all trees

#3 diabetes or diabetic:ti,ab

#4 #1 OR #2 OR #3

#5 MeSH descriptor: [Community-Acquired Infections] explode all trees

#6 MeSH descriptor: [Pneumonia, Bacterial] explode all trees

#7 MeSH descriptor: [Pneumonia, Viral] explode all trees

#8 community-acquired or CAP:ti,ab

#9 #5 OR #6 OR #7 OR #8

#10 epidemiology or incidence or prevalence or mortality or pathophysiology or etiology or microbiology or pathogen* or treatment or therapy or prevention or vaccine*:ti,ab

#11 #4 AND #9 AND #10

#12 hospital-acquired or nosocomial or healthcare-associated:ti,ab

#13 #11 NOT #12

Search limited to Adult (≥19 y) and English.

Database: Web of Science (Core Collection)

TS = ((diabetes OR diabetic) AND (community-acquired OR CAP) AND (epidemiology OR incidence OR prevalence OR mortality OR pathophysiology OR etiology OR microbiology OR pathogen* OR treatment OR therapy OR prevention OR vaccine*))

NOT TS = (hospital-acquired OR nosocomial OR healthcare-associated)

Refined by: English, Humans, Adult (19+ years), Time span 1999-08-31 to 2025-08-31.
